# A diaphragmatic electrical activity-based optimization strategy during pressure support ventilation improves synchronization but does not impact work of breathing

**DOI:** 10.1186/s13054-017-1599-z

**Published:** 2017-01-31

**Authors:** Francois Beloncle, Lise Piquilloud, Nuttapol Rittayamai, Christer Sinderby, Hadrien Rozé, Laurent Brochard

**Affiliations:** 1 Interdepartmental Division of Critical Care Medicine, University of Toronto, Toronto, ON Canada; 2 Keenan Research Centre and Li Ka Shing Knowledge Institute, St. Michael’s Hospital, 30 Bond St, Toronto, ON M5B 1W8 Canada; 3 Medical Intensive Care Unit, Hospital of Angers, University of Angers, Angers, France; 4 Adult Intensive Care and Burn Unit, University Hospital of Lausanne (CHUV), Lausanne, Switzerland; 5 Division of Respiratory Diseases and Tuberculosis, Department of Medicine, Faculty of Medicine Siriraj Hospital, Bangkok, Thailand; 6 CHU de Bordeaux, Service d’Anesthesie-Reanimation 2, Pessac, 33600 France

**Keywords:** Mechanical ventilation, Pressure support ventilation, Neurally adjusted ventilatory assist, Chronic pulmonary obstructive, Restrictive disease, Asynchrony

## Abstract

**Background:**

Poor patient-ventilator synchronization is often observed during pressure support ventilation (PSV) and has been associated with prolonged duration of mechanical ventilation and poor outcome. Diaphragmatic electrical activity (Eadi) recorded using specialized nasogastric tubes is a surrogate of respiratory brain stem output. This study aimed at testing whether adapting ventilator settings during PSV using a protocolized Eadi-based optimization strategy, or Eadi-triggered and -cycled assisted pressure ventilation (or PSV_N_) could (1) improve patient-ventilator interaction and (2) reduce or normalize patient respiratory effort as estimated by the work of breathing (WOB) and the pressure time product (PTP).

**Methods:**

This was a prospective cross-over study. Patients with a known chronic pulmonary obstructive or restrictive disease, asynchronies or suspected intrinsic positive end-expiratory pressure (PEEP) who were ventilated using PSV were enrolled in the study. Four different ventilator settings were sequentially applied for 15 minutes (step 1: baseline PSV as set by the clinician, step 2: Eadi-optimized PSV to adjust PS level, inspiratory trigger, and cycling settings, step 3: step 2 + PEEP adjustment, step 4: PSV_N_). The same settings as step 3 were applied again after step 4 to rule out a potential effect of time. Breathing pattern, trigger delay (T_d_), inspiratory time in excess (T_iex_), pressure-time product (PTP), and work of breathing (WOB) were measured at the end of each step.

**Results:**

Eleven patients were enrolled in the study. Eadi-optimized PSV reduced T_d_ without altering T_iex_ in comparison with baseline PSV. PSV_N_ reduced T_d_ and T_iex_ in comparison with baseline and Eadi-optimized PSV. Respiratory pattern did not change during the four steps. The improvement in patient-ventilator interaction did not lead to changes in WOB or PTP.

**Conclusions:**

Eadi-optimized PSV allows improving patient ventilator interaction but does not alter patient effort in patients with mild asynchrony.

**Trial registration:**

Clinicaltrials.gov identifier: NCT 02067403. Registered 7 February 2014.

**Electronic supplementary material:**

The online version of this article (doi:10.1186/s13054-017-1599-z) contains supplementary material, which is available to authorized users.

## Background

Pressure support ventilation (PSV) is well tolerated [1] and has been helpful to reduce both adverse effects of prolonged sedation [2] and ventilator-associated diaphragmatic dysfunction [3, 4]. Thus, it is widely used as soon as deep sedation and/or muscle paralysis and controlled mechanical ventilation are no longer required to oxygenate the patient. During PSV, each ventilator-delivered cycle is initiated by a flow or pressure variation (pneumatic signal) resulting from the patient’s inspiratory effort. Pressurization is then delivered by the ventilator and lasts until a predetermined flow-based cycling-off criterion is reached. The amount of pressure delivered by the ventilator under PSV is constant and set by the clinician [5]. During PSV, the work of breathing (WOB) is shared between the patient and the ventilator. Due to differences in patients’ and ventilators’ respiratory profiles [6, 7], poor patient-ventilator synchronization is often observed [8, 9] and has been associated with abnormal WOB [10] and prolonged duration of mechanical ventilation [8, 11–13]. A poor synchronization has been associated with suboptimal ventilator settings, especially over-assist [14], non-optimized expiratory cycling [7], and positive end-expiratory pressure (PEEP) setting [12, 15].

At the bedside, as no visual information on the patient’s inspiratory activity is usually available on the ventilator screen, detecting patient-ventilator asynchronies is sometimes difficult even for experienced clinicians [16]. Diaphragmatic electrical activity (Eadi), used as a surrogate of respiratory brain stem output, recorded using specialized nasogastric tubes equipped with electrodes, can simplify the detection of patient-ventilator asynchronies and could be used to optimize the ventilator settings to improve the matching between the patient and the ventilator. Eadi signal can also be used in patients to deliver an assisted ventilation, synchronized and proportional to patient demand (NAVA mode) [17, 18]. This study aimed at testing whether adapting ventilator settings (inspiratory trigger sensitivity, pressure support level, cycling-off criterion, and PEEP) during PSV using a protocolized Eadi-based optimization strategy or Eadi-triggered and -cycled assisted pressure ventilation (or neural PSV, PSV_N_) could (1) improve patient-ventilator interaction and (2) modify patient’s respiratory effort as estimated by the work of breathing (WOB) and the pressure time product (PTP) of the respiratory muscles in comparison with standard PSV.

## Methods

This was a prospective cross-over study. It took place in the medical/surgical ICU of St Michael’s hospital in Toronto, Canada, from March to October 2014. The trial was registered at clinicaltrials.gov (NCT 02067403).

### Patients

Patients who were ventilated using PSV for an expected duration of ventilation of more than 24 hours and who had a known or suspected history of chronic pulmonary obstructive (COPD) or restrictive disease, obesity (defined as body mass index (BMI) ≥ 30 kg.m^-2^), visible asynchronies or suspected intrinsic PEEP, were enrolled in the study. Exclusion criteria were contraindication to nasogastric tube placement, poor short-term prognosis or “Do not resuscitate” order already established and in palliative care.

### Data acquisition/physiological measurements

At study inclusion, patients’ demographic and medical characteristics, arterial blood gas analysis, Sequential Organ Failure Assessment (SOFA) score, Acute Physiology and Chronic Health Evaluation III (APACHE III) score, and baseline ventilator settings were recorded.

A specific nasogastric tube (Eadi catheter) equipped with electrodes and an esophageal balloon (Neurovent, Toronto, ON, Canada) was inserted. The Eadi catheter was connected to a Servo-I ventilator (Maquet, Solna, Sweden). Its position was controlled on the ventilator screen according to the manufacturer’s instructions and previously published studies [19]. The calibration procedure of esophageal pressure (Pes) consisted of an occlusion test (or Baydur maneuver) (two to five inspiratory efforts) [20, 21].

A personal computer was connected to the ventilator. Flow, airway pressure (Paw), and Eadi waveforms were acquired from the ventilator using a dedicated software with a sampling frequency of 100 Hz (ServoTracker, Maquet, Solna, Sweden). Pes and Paw (measured between the Y piece of the ventilator circuit and the endotracheal tube) were recorded at 100 Hz by an analog/numeric data-acquisition system (MP150, Biopac Systems, Goleta, CA, USA) connected to a second personal computer. All the aforementioned waveforms were continuously recorded for 5 minutes after a stabilization period of 10 minutes and were secondarily synchronized for offline analysis. Briefly, we synchronized both files to get the 0 flow point of the same respiratory cycle perfectly matched.

Trigger delay (T_d_) was defined as the time difference between the initial increase in Eadi (visually determined) and the beginning of the ventilator-delivered pressurization. Inspiratory time in excess (T_iex_) was calculated as the time difference between the time when Eadi decreased to 70% of peak Eadi and the end of ventilator-delivered pressurization (Additional file 1).

Five types of major patient-ventilator asynchronies (autotriggering, ineffective effort, double triggering, delayed cycling and premature cycling) as defined by Thille et al. [8] were determined by visual analysis from airway pressure, flow and Eadi curves over the 5 minutes recording period. Additionally, we computed during PSV_N_the number of pseudo-autotriggerings defined as a significant pressurization delivered by the ventilator (>50% of PEEP level) not related to a patient’s effort [22]. Example of pseudo-autotriggerings is represented in Additional file 2.

The global asynchrony index was computed as the sum of the five types of major asynchronies but not pseudo-autotriggerings. Severe asynchrony was defined as a global asynchrony index greater than 10% [12, 23].

The neuroventilatory efficiency (NVE) expresses the ability to generate volume normalized to neural drive and was defined as the ratio of tidal volume (Vt) over peak of the Eadi (Eadi_max_).

A semi-automated research software, described in previous works [24] was used for WOB and PTP measurements (SR program, non-commercially available, Barcelona, Spain).

WOB was determined from esophageal pressure measurement using the Campbell diagram as previously described [25].

PTP was obtained by measuring the area under the esophageal pressure signal between the onset of the inspiratory effort and the end of inspiration, defined as the end of inspiratory flow signal. This area was referenced to the chest wall static recoil pressure-time curve relationship [26].

For each step, respiratory rate (RR), tidal volume (Vt), minute ventilation, T_d_, T_iex_, Eadi_max_, area under the curve of the Eadi (Eadi_AUC_), NVE, WOB and PTP were measured for the 25 initial breathing cycles during the recording period and were averaged.

### Study protocol

Once the specific nasogastric tube was correctly positioned, four different ventilator settings corresponding to five sequential steps were applied for 10 minutes, followed by a recording period of 5 minutes for all conditions.

At inclusion, patients were ventilated with PSV as set by the attending physician and respiratory therapists in charge of the patient in order to target a respiratory rate between 20 and 30/minute and with a PEEP setting ≥ 5 cmH2O (step 1).

Asynchronies were screened at the bedside using Paw, flow, and Eadi curves. The T_d_ and T_iex_ were estimated by freezing the screen and using cursors. During step 2, Eadi monitoring was used to sequentially optimize PS level, inspiratory trigger, and cycling settings to optimize patient-ventilator synchrony. In more detail, if ineffective efforts were observed, first the sensitivity of the inspiratory trigger was adapted to the lowest possible value without inducing auto-triggerings. Then, pressure support level was decreased as low as possible without inducing significant tachypnoea until ineffective efforts disappeared. Third, cycling-off criterion was gradually adjusted to decrease T_iex_, based on Eadi signal visualization. If premature cyclings and/or double triggerings were present, insufflation time was gradually increased by decreasing the cycling-off criterion. During step 3a, Eadi signal was also used to adapt PEEP setting. Practically, if a prolonged T_d_ was observed, PEEP was increased by a step of 1 cm H_2_O until T_d_ did not further decrease, up to a maximal value of 12 cmH_2_O. After this titration process, the PEEP value was selected as the lowest PEEP corresponding to the lowest T_d_. During step 4, the ventilator was switched to PSV_N_. PSV_N_ consisted of using the advantage of the triggering and cycling function of the neurally adjusted ventilatory assist (NAVA) mode but limiting the pressure to the same level than during PSV and using a high NAVA gain to create a square pressure wave. NAVA mode ventilation was thus set with the highest gain level (15 cmH_2_O/μV) to provide very fast pressurization mimicking pressure support pressurization shape, to better match the initial inspiratory demand, which can be particularly high in the presence of respiratory distress [27, 28]. The advantage of this mode would be to look very similar to clinicians used to pressure support ventilation but with an improved synchrony. The pressure limit was chosen to get the same level of assistance between step 3a and 4.

The level of PEEP during step 4 was the same as during step 3a. After termination of step 4, the same settings as step 3a were applied again to rule out a potential effect of time (Step 3b).

### Statistical analysis

As no previously published data allowed quantifying a benefit, no sample size calculation could be performed in this physiological study. Nonparametric tests were used because of the small number of patients. Sequentially, for each parameter, the absence of difference between steps 3a and 3b were verified using Wilcoxon tests. The results of steps 3a and 3b were considered together and the average values of the two steps are presented as step 3. The measured parameters were compared across the different steps using nonparametric Friedman test. Wilcoxon tests were used to perform post hoc pairwise comparisons with correction for multiple comparisons using the false discovery rate approach. Statistical significance was defined as *p* value <0.05. The statistical analysis was performed using Prism (GraphPad Software v5.0b, La Jolla, CA, USA).

## Results

### Patient characteristics

The study included 11 patients. The main characteristics at inclusion are detailed in Table 1. Six (55%) patients had a medical history of chronic obstructive pulmonary disease (COPD).

**Table 1:** Patient characteristics at inclusion

Parameters	11 patients
Age (years)	70 (68–80)
Sex M/F	7/4
Body mass index (kg.m^-2^)	25.4 (22–30)
Comorbidities	
COPD	6 (55%)
Obesity	5 (45%)
Bronchiectasis	1 (9%)
LV dysfunction	1 (9%)
Interstitial pulmonary disease	1 (9%)
APACHE III	20 (17–23.5)
SOFA score total at inclusion	5 (3.5–8)
respiratory	2 (1.5–3)
cardiovascular	1 (0–2)
neurologic	0.5 (0–1)
hepatic	0 (0–0)
hematologic	0 (0–1)
renal	1 (0–1.5)
ICU admission diagnosis	Acute respiratory failure 5 (45%)
	Stroke 2 (18%)
	Postoperative 1 (9%)
	Gastrointestinal bleeding 1 (9%)
	Cardiac arrest 1 (9%)
	Trauma 1 (9%)
Days from ICU admission	9 (2–15)
Days from initiation of mechanical ventilation	4 (1–13)
pH	7.36 (7.32–7.39)
PaO_2_ (mmHg)	102.5 (83–133)
FiO_2_	0.5 (0.4–0.5)
PaO_2_/FiO_2_ (mmHg)	205 (165–338)
PaCO_2_ (mmHg)	47 (37.5–51.5)
HCO_3_- (mmHg)	29 (22.5–30.5)

Results are presented as median and interquartile range or number and percentage

*COPD* chronic obstructive pulmonary disease, *LV dysfunction* left ventricular dysfunction, *SOFA* Sepsis-related Organ Failure Assessment, *APACHE III* Acute Physiology and Chronic Health Evaluation III

### Changes in ventilator settings during the study

Ventilator settings during the four steps are mentioned in Table 2. Pressure support level was never modified. The inspiratory trigger sensitivity was increased in step 2 in comparison with step 1 in five patients (45%). It was not modified in six patients (55%). Cycling-off criterion was higher in step 2 than in step 1 in eight patients (73%), was lower in one patient (9%) and was not modified in two patients (18%). PEEP level was increased from step 2 to step 3 in four patients (36%) and was not modified in seven patients (64%).

**Table 2:** Ventilator settings during the four steps

	Step 1	Step 2	Step 3	Step 4	*p*
Inspiratory trigger sensitivity (flow trigger)	2 (2–2)	3 (2–4)	3 (2–4)	-	0.004
PEEP (cmH_2_0)	8 (5.75–8)	8 (5.75–8)	8 (7.25–9.75)	8 (7.25–9.75)	0.007
PS level (cmH_2_0)	9 (8–10)	9 (8–10)	9 (8–10)	-	-
Cycling criterion (%)	30 (30–30)	47.5 (40–53.75)	47.5 (40–53.75)	-	<0.001

Results are presented as median and interquartile range

*PEEP* positive end expiratory pressure, *PS* pressure support

### Effect of PSV optimization and PSV_N_ on synchronization

**Fig. 1:**
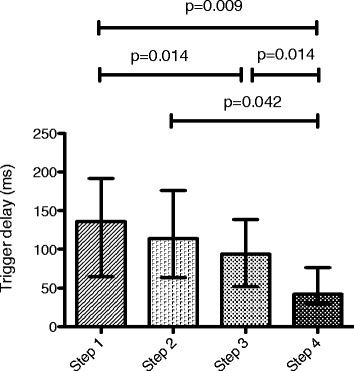
Trigger delay during the four steps. Plots represent median and interquartile range (overall comparison, *p* = 0.002)

Eadi-optimized PSV (step 3) allowed reducing T_d_ in comparison with standard PSV (step 1) (Fig. 1 and Additional file 3). The T_d_ was shorter in PSV_N_ (step 4) than in the other steps using baseline or optimized PSV mode (Steps 1, 2, 3).

**Fig. 2:**
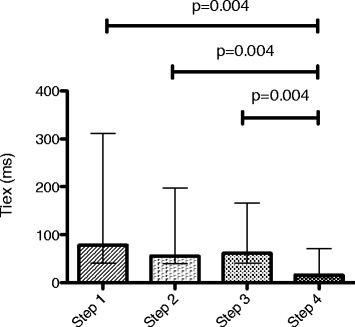
Inspiratory time in excess (T_iex_) during the four steps. Plots represent median and interquartile range (overall comparison, *p* = 0.014)

Eadi PSV optimization did not modify T_iex_ (Fig. 2 and Additional file 3). However T_iex_ was reduced during PSV_N_ in comparison with baseline and optimized PSV mode.

Patients had very few asynchrony events. The global asynchrony indexes were not different during the four steps ((1 (0–1.5) %; 0 (0–2.5) %; 0 (0–1) %; and 0 (0–4) % in steps 1, 2, 3, and 4, respectively). Of note, two patients had a high incidence of asynchronies in PSV_N_ (global asynchrony index of 10 and 12% respectively) due to double triggerings (of unclear clinical significance), whereas they presented a very low incidence of asynchronies in baseline and Eadi-optimized PSV. Frequent pseudo-autotriggering were also observed in PSV_N_ (pseudo-autotriggering asynchrony index of 4 (0.5–8.5) %). By contrast, one patient with severe restrictive disease presented severe asynchrony due to premature cyclings and double triggerings in steps 1, 2, and 3 (79, 24, and 41% respectively) but did not present any asynchrony event in step 4 (Additional file 4).

### Effect of PSV optimization and PSV_N_ on breathing pattern, Eadi, and NVE

Breathing pattern (RR, Vt, and minute ventilation), Eadi (peak and area under the curve of the Eadi) and NVE were not altered by ventilator settings modifications (Table 3).

**Table 3:** Breathing pattern, electrical activity of the diaphragm (Eadi), and neuroventilatory efficiency (NVE) during the four steps

	Step 1	Step 2	Step 3	Step 4	*p*
Respiratory rate (breaths.min^-1^)	22 (18–29)	20 (19–28)	23 (19–26)	20 (19–26)	0.679
Tidal volume (mL)	432 (340–521)	419 (336–586)	442 (332–582)	440 (340–576)	0.66
Minute ventilation (L.min^-1^)	9.0 (7.7–11.1)	9.6 (7.4–11.3)	9.6 (7.1–11.8)	9.5 (7.5–10.9)	0.792
Eadi_max_ (μV)	18.4 (10.5–25.6)	18.0 (12.6–21.4)	21.5 (14.5–23.4)	16.8 (14.0–22.3)	0.819
Eadi_AUC_ (μV.s)	11.6 (6.7–17.9)	9.9 (8.6–13.5)	13.9 (9.1–16.4)	11.5 (8.4–14.1)	0.819
NVE (mL.μV^-1^)	30.5 (12.0–45.3)	23.7 (18.6–39.3)	23.4 (17.6–36.8)	28.5 (19.0–36.7)	0.819

Results are presented as median and interquartile range

*Eadi*_*max*_ peak of the Eadi, *Eadi*_*AUC*_ area under the curve of the Eadi, *NVE* neuroventilatory efficiency

### Effect of PSV optimization and PSV_N_ on patients’ effort

**Fig. 3:**
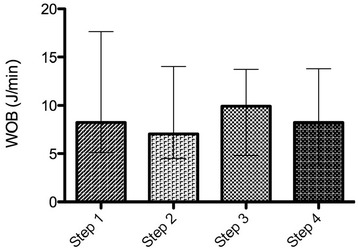
Work of breathing (WOB) during the four steps. Plots represent median and interquartile range (overall comparison, *p* = 0.301)

**Fig. 4:**
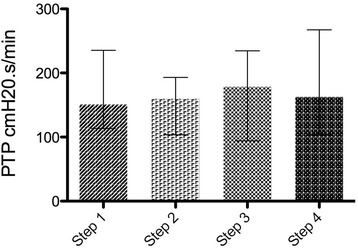
Pressure time product (PTP) during the four steps. Plots represent median and interquartile range (overall comparison, *p* = 0.126)

The improvement in patient-ventilator interaction was not associated with changes in WOB (Fig. 3 and Additional file 5) or PTP (Fig. 4 and Additional file 5).

## Discussion

The present study showed in a population of patients with few major asynchronies when ventilated using standard PSV that an Eadi-based optimization strategy allowed improving patient-ventilator interaction but with minimal changes overall in breathing pattern or effort. More specifically, Eadi-optimized PSV reduced T_d_ whereas Eadi-triggered and -cycled pressure assist ventilation (PSV_N_) further reduced T_d_ and optimized expiratory synchrony. Thus these new alternatives to ventilate patients combine the advantage of Eadi-based trigger and cycling-off in terms of patient-ventilator synchrony and the advantage of standard PSV in terms of pressurization and clinical use for most clinicians familiar to PSV. There was no concomitant measurable change in patient effort.

Before further discussing the results, several limitations should be addressed. First, this physiological study only included 11 patients. These patients had only few major asynchronies suggesting that the ventilator settings at baseline were already well adapted and thus difficult to further optimize in terms of major asynchronies. Second, only a short period of time was observed and it cannot be excluded that different results could have been demonstrated if a prolonged period of time had been studied. However this allowed keeping patients stable enough to compare the different steps. This was demonstrated by performing step 3b to rule out a time effect. Third, the offline synchrony analysis was performed manually and can sometimes be prone to interpretation. However, the reading methodology followed well-defined previously published criteria.

Optimized PSV was obtained during steps 2 and 3 using information regarding the intensity and timing of inspiratory demand coming from the brain stem respiratory centers but recorded in periphery as Eadi. Theoretically, using this information on a real-time basis should allow delivering assisted ventilation synchronized to patient demand. During step 2 Eadi was used to monitor asynchronies and to optimize ventilator settings to reduce patient-ventilator asynchronies as much as possible. As a low level of assist was set by the clinicians already trained to avoid over-assist, there were virtually no patient-ventilator major asynchronies at baseline and no pressure support level adaptation was required. By allowing seeing the duration of inspiration (and thus T_iex_) at the bedside during PSV, Eadi monitoring could be used to optimize the expiratory trigger, a setting often difficult to optimize in daily practice but closely related to the occurrence of asynchronies when not optimally set [7, 29]. Practically, in our study Eadi information led to expiratory trigger threshold changes in 82% of the subjects but did not alter T_iex_. Eadi was also used to optimize PEEP setting during step 3, another challenge at the bedside especially during PSV.

Eadi-triggered and -cycled assisted pressure ventilation (or PSV_N_) reduces T_d_ in comparison with both standard PSV and Eadi-optimized PSV mode using pneumatic signal. These results are consistent with previous studies performed in standard NAVA mode [17, 22, 30] or in PSV_N_ mode during invasive ventilation [31] or during helmet-delivered noninvasive ventilation [32, 33] and were expected, as an Eadi increase is detected earlier by the ventilator than flow or pressure changes [34] related to air motion in the lungs. Interestingly, the very short T_d_ observed in PSV_N_ (<50 ms) is lower than the conscious threshold of perception (about 150 ms) [35], which may contribute to optimize patient comfort. In our study, as in Liu et al. data [31], PSV_N_ allows improving T_iex_ in comparison with standard and Eadi-optimized PSV mode.

However, during PSV_N_, pseudo-autotriggerings due to very small diaphragm contraction or Eadi signal artefacts were frequent and may have led to patient discomfort. This may be related to the very high amplification provided by PSV_N_.

Despite the improvement in patient-ventilator interaction, WOB and PTP were not altered in Eadi-optimized PSV or PSV_N_. This may be explained by the low pressure support level and the low number of major asynchronies observed in the four steps. An Eadi-based optimization strategy is more likely to impact patient effort in patients with greater assistance and poorer patient-ventilator synchrony. In addition, our study illustrates that individualization of the approach is necessary since results in patients with restrictive, obstructive or obesity-associated disease may need different settings. Of note, Passath et al. demonstrated that an increase in PEEP was associated with a decrease in Eadi for large PEEP variations [36]. We did not observe any changes in Eadi when we increased PEEP in our study (step 3). This difference may be explained by the lower range of PEEP variation in our study.

Eadi-optimized PSV and PSV_N_ allowed better patient-ventilator synchrony and rapid pressurization. The major advantage of using the Eadi signal in these ways is to optimize a mode of ventilation well known and familiar to all clinicians.

## Conclusions

Even if the interest of optimizing T_d_ and T_iex_ can be challenged from a clinical point of view in the absence of major asynchronies reduction, this study demonstrates the feasibility and interest of using Eadi recording to optimize patient ventilator interaction. Based on this result, outcome studies to assess the interest of using this strategy by default are now required before recommending this approach.

## Additional files

Additional file 1: Definition of trigger delay (T_d_) and inspiratory time in excess (T_iex_). Paw, airway pressure; Eadi, electrical activity of the diaphragm. (PDF 63 kb)
Additional file 2: Example of pseudo-autotriggering (*arrow*) in Eadi-triggered and cycle- assisted pressure ventilation (or PSV_N_) mode. Paw, airway pressure; Pes, esophageal pressure; Eadi, electrical activity of the diaphragm. Pseudo-autotriggerings are defined as a significant pressurization delivered by the ventilator not related to a patient’s effort. Note the absence of deflection in esophageal pressure demonstrating the absence of patient effort. (PDF 60 kb)
Additional file 3: Trigger delay (A) and inspiratory time in excess (T_iex_) (B) during the four steps. Individual data. *Horizontal red lines* represent the median values. (PDF 45 kb)
Additional file 4: Airway pressure (Paw), flow, esophageal pressure (Pes) and electrical activity of the diaphragm (Eadi) tracings during the four steps in a restrictive patient. A, step 1; B, step 2; C, step 3 and D, step 4. (PDF 261 kb)
Additional file 5: Work of breathing (WOB) (A) and pressure time product (PTP) (B) during the four steps. Individual data. *Horizontal red lines* represent the median values. (PDF 45 kb)

## Abbreviations

COPD: Chronic obstructive pulmonary disease
Eadi: Electrical activity of the diaphragm
Eadi_AUC_: Area under the curve of the Eadi
Eadi_max_: Peak of the Eadi
NAVA: Neurally adjusted ventilatory assist
NVE: Neuroventilatory efficiency
Paw: Airway pressure
PEEP: Positive end-expiratory pressure
Pes: Esophageal pressure
PSV: Pressure support ventilation
PSV_N_: Neural pressure support ventilation
PTP: Pressure-time product
T_D_: Trigger delay
T_iex_: Inspiratory time in excess
Vt: Tidal volume
WOB: Work of breathing

## References

[1] Russell WC, Greer JR (2000). The comfort of breathing: a study with volunteers assessing the influence of various modes of assisted ventilation. Crit Care Med.

[2] Girard TD, Kress JP, Fuchs BD, Thomason JWW, Schweickert WD, Pun BT (2008). Efficacy and safety of a paired sedation and ventilator weaning protocol for mechanically ventilated patients in intensive care (Awakening and Breathing Controlled trial): a randomised controlled trial. Lancet.

[3] Jaber S, Jung B, Matecki S, Petrof BJ (2011). Clinical review: ventilator-induced diaphragmatic dysfunction--human studies confirm animal model findings!. Crit Care Lond Engl.

[4] Futier E, Constantin J-M, Combaret L, Mosoni L, Roszyk L, Sapin V (2008). Pressure support ventilation attenuates ventilator-induced protein modifications in the diaphragm. Crit Care Lond Engl.

[5] MacIntyre N, Nishimura M, Usada Y, Tokioka H, Takezawa J, Shimada Y (1990). The Nagoya conference on system design and patient-ventilator interactions during pressure support ventilation. Chest.

[6] Tobin MJ, Jubran A, Laghi F (2001). Patient-ventilator interaction. Am J Respir Crit Care Med.

[7] Yamada Y, Du HL (2000). Analysis of the mechanisms of expiratory asynchrony in pressure support ventilation: a mathematical approach. J Appl Physiol.

[8] Thille AW, Rodriguez P, Cabello B, Lellouche F, Brochard L (2006). Patient-ventilator asynchrony during assisted mechanical ventilation. Intensive Care Med.

[9] Doorduin J, Sinderby CA, Beck J, van der Hoeven JG, Heunks LMA (2014). Automated patient-ventilator interaction analysis during neurally adjusted non-invasive ventilation and pressure support ventilation in chronic obstructive pulmonary disease. Crit Care Lond Engl.

[10] Kondili E, Prinianakis G, Georgopoulos D (2003). Patient-ventilator interaction. Br J Anaesth.

[11] De Wit M, Pedram S, Best AM, Epstein SK (2009). Observational study of patient-ventilator asynchrony and relationship to sedation level. J Crit Care.

[12] Chao DC, Scheinhorn DJ, Stearn-Hassenpflug M (1997). Patient-ventilator trigger asynchrony in prolonged mechanical ventilation. Chest.

[13] Blanch L, Villagra A, Sales B, Montanya J, Lucangelo U, Luján M (2015). Asynchronies during mechanical ventilation are associated with mortality. Intensive Care Med.

[14] Thille AW, Cabello B, Galia F, Lyazidi A, Brochard L (2008). Reduction of patient-ventilator asynchrony by reducing tidal volume during pressure-support ventilation. Intensive Care Med.

[15] Nava S, Bruschi C, Rubini F, Palo A, Iotti G, Braschi A (1995). Respiratory response and inspiratory effort during pressure support ventilation in COPD patients. Intensive Care Med.

[16] Colombo D, Cammarota G, Alemani M, Carenzo L, Barra FL, Vaschetto R (2011). Efficacy of ventilator waveforms observation in detecting patient-ventilator asynchrony. Crit Care Med.

[17] Piquilloud L, Vignaux L, Bialais E, Roeseler J, Sottiaux T, Laterre P-F (2011). Neurally adjusted ventilatory assist improves patient-ventilator interaction. Intensive Care Med.

[18] Demoule A, Clavel M, Rolland-Debord C, Perbet S, Terzi N, Kouatchet A (2016). Neurally adjusted ventilatory assist as an alternative to pressure support ventilation in adults: a French multicentre randomized trial. Intensive Care Med.

[19] Carteaux G, Córdoba-Izquierdo A, Lyazidi A, Heunks L, Thille AW, Brochard L (2016). Comparison between neurally adjusted ventilatory assist and pressure support ventilation levels in terms of respiratory effort. Crit Care Med.

[20] Baydur A, Behrakis PK, Zin WA, Jaeger M, Milic-Emili J (1982). A simple method for assessing the validity of the esophageal balloon technique. Am Rev Respir Dis.

[21] Akoumianaki E, Maggiore SM, Valenza F, Bellani G, Jubran A, Loring SH (2014). The application of esophageal pressure measurement in patients with respiratory failure. Am J Respir Crit Care Med.

[22] Piquilloud L, Tassaux D, Bialais E, Lambermont B, Sottiaux T, Roeseler J (2012). Neurally adjusted ventilatory assist (NAVA) improves patient-ventilator interaction during non-invasive ventilation delivered by face mask. Intensive Care Med.

[23] Vitacca M, Bianchi L, Zanotti E, Vianello A, Barbano L, Porta R (2004). Assessment of physiologic variables and subjective comfort under different levels of pressure support ventilation. Chest.

[24] L’Her E, Deye N, Lellouche F, Taille S, Demoule A, Fraticelli A (2005). Physiologic effects of noninvasive ventilation during acute lung injury. Am J Respir Crit Care Med.

[25] Cabello B, Mancebo J (2006). Work of breathing. Intensive Care Med.

[26] Sassoon CS, Light RW, Lodia R, Sieck GC, Mahutte CK (1991). Pressure-time product during continuous positive airway pressure, pressure support ventilation, and T-piece during weaning from mechanical ventilation. Am Rev Respir Dis.

[27] Bonmarchand G, Chevron V, Chopin C, Jusserand D, Girault C, Moritz F (1996). Increased initial flow rate reduces inspiratory work of breathing during pressure support ventilation in patients with exacerbation of chronic obstructive pulmonary disease. Intensive Care Med.

[28] Bonmarchand G, Chevron V, Ménard JF, Girault C, Moritz-Berthelot F, Pasquis P (1999). Effects of pressure ramp slope values on the work of breathing during pressure support ventilation in restrictive patients. Crit Care Med.

[29] Tassaux D, Gainnier M, Battisti A, Jolliet P (2005). Impact of expiratory trigger setting on delayed cycling and inspiratory muscle workload. Am J Respir Crit Care Med.

[30] Spahija J, de Marchie M, Albert M, Bellemare P, Delisle S, Beck J (2010). Patient-ventilator interaction during pressure support ventilation and neurally adjusted ventilatory assist. Crit Care Med.

[31] Liu L, Xia F, Yang Y, Longhini F, Navalesi P, Beck J (2015). Neural versus pneumatic control of pressure support in patients with chronic obstructive pulmonary diseases at different levels of positive end expiratory pressure: a physiological study. Crit Care Lond Engl.

[32] Moerer O, Beck J, Brander L, Costa R, Quintel M, Slutsky AS (2008). Subject-ventilator synchrony during neural versus pneumatically triggered non-invasive helmet ventilation. Intensive Care Med.

[33] Cammarota G, Longhini F, Perucca R, Ronco C, Colombo D, Messina A (2016). New setting of neurally adjusted ventilatory assist during noninvasive ventilation through a helmet. Anesthesiology.

[34] Sinderby C, Navalesi P, Beck J, Skrobik Y, Comtois N, Friberg S (1999). Neural control of mechanical ventilation in respiratory failure. Nat Med.

[35] Whitelaw WA, Derenne JP, Milic-Emili J (1975). Occlusion pressure as a measure of respiratory center output in conscious man. Respir Physiol.

[36] Passath C, Takala J, Tuchscherer D, Jakob SM, Sinderby C, Brander L (2010). Physiologic response to changing positive end-expiratory pressure during neurally adjusted ventilatory assist in sedated, critically ill adults. Chest.

