# Supplementary figures and images for: A diaphragmatic electrical activity-based optimization strategy during pressure support ventilation improves synchronization but does not impact work of breathing
Source: Crit Care. 2017 Jan 31;21:21. doi: 10.1186/s13054-017-1599-z (PMC5282691; doi:10.1186/s13054-017-1599-z)

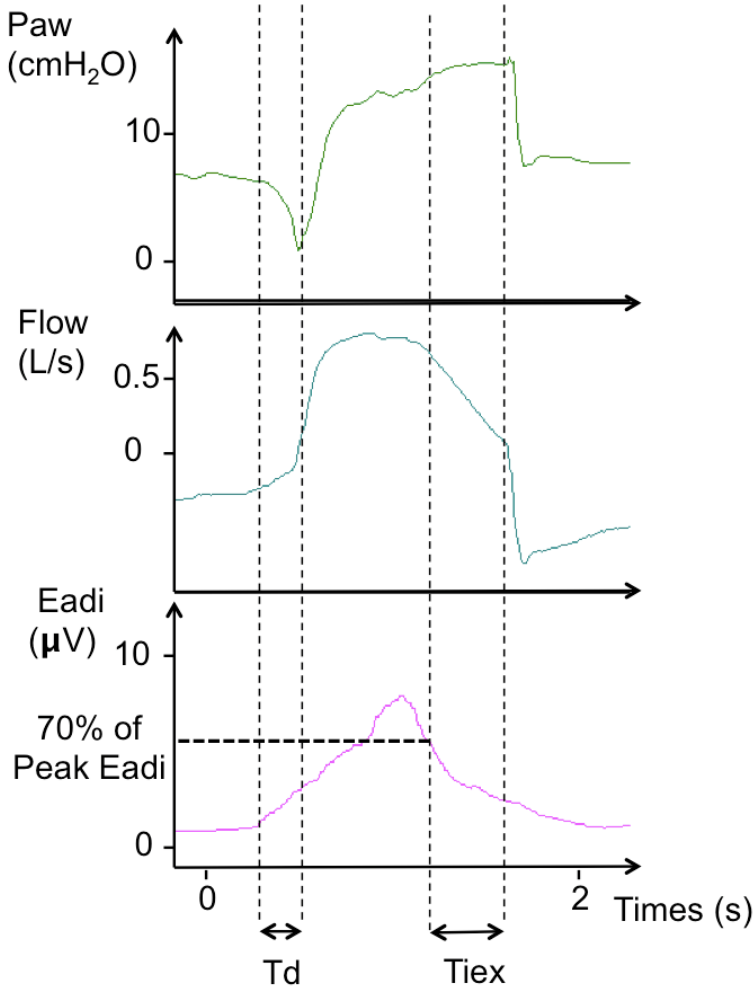

Supplement: Additional file 1: — Definition of trigger delay (Td) and inspiratory time in excess (Tiex). Paw, airway pressure; Eadi, electrical activity of the diaphragm. (PDF 63 kb) [file 13054_2017_1599_MOESM1_ESM.pdf]

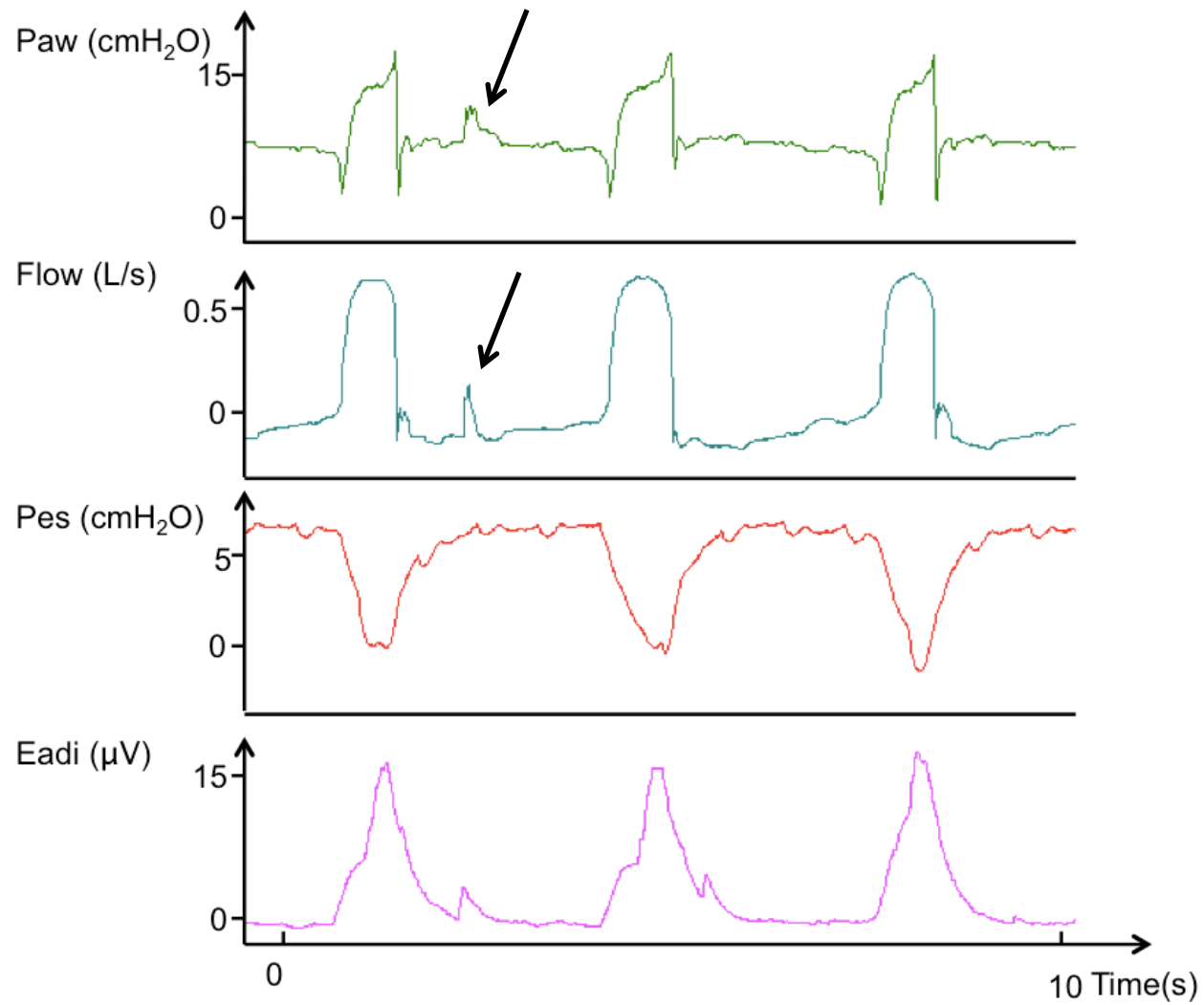

Supplement: Additional file 2: — Example of pseudo-autotriggering (arrow) in Eadi-triggered and cycle- assisted pressure ventilation (or PSVN) mode. Paw, airway pressure; Pes, esophageal pressure; Eadi, electrical activity of the diaphragm. Pseudo-autotriggerings are defined as a significant pressurization delivered by the ventilator not related to a patient’s effort. Note the absence of deflection in esophageal pressure demonstrating the absence of patient effort. (PDF 60 kb) [file 13054_2017_1599_MOESM2_ESM.pdf]

**A**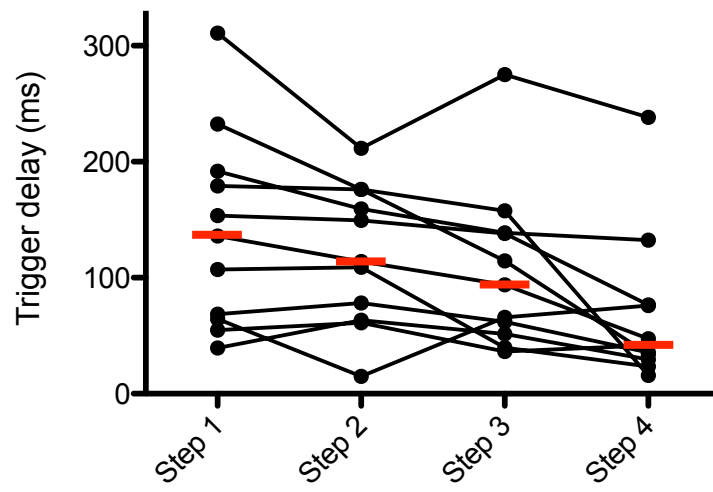**B**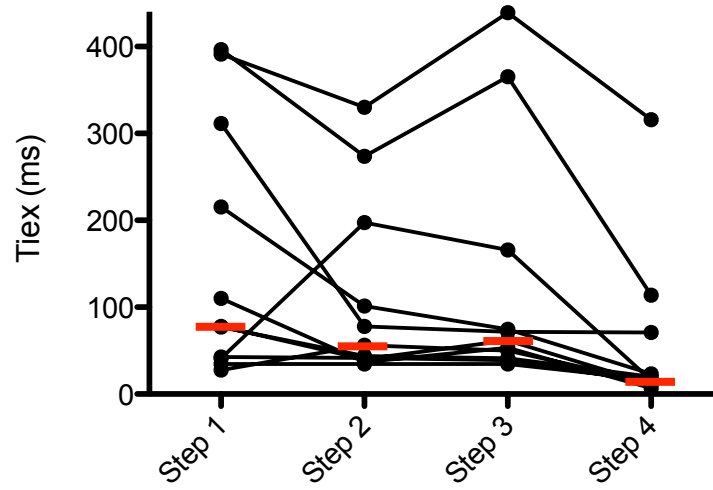

Supplement: Additional file 3: — Trigger delay (A) and inspiratory time in excess (Tiex) (B) during the four steps. Individual data. Horizontal red lines represent the median values. (PDF 45 kb) [file 13054_2017_1599_MOESM3_ESM.pdf]

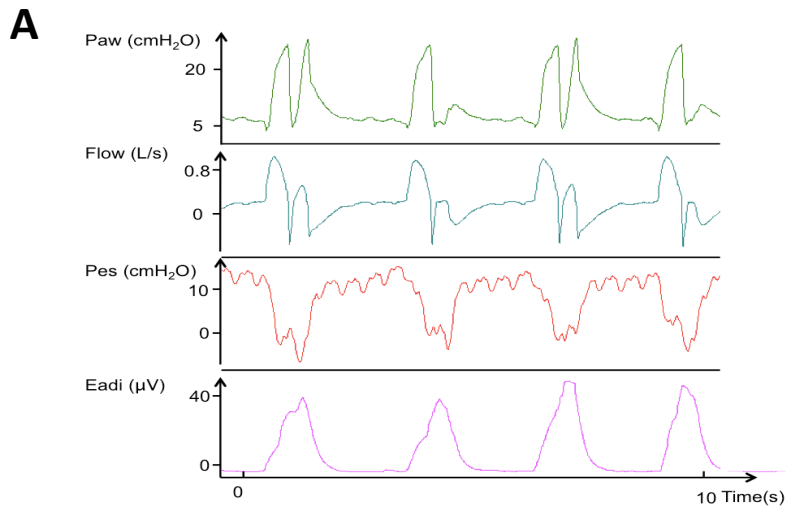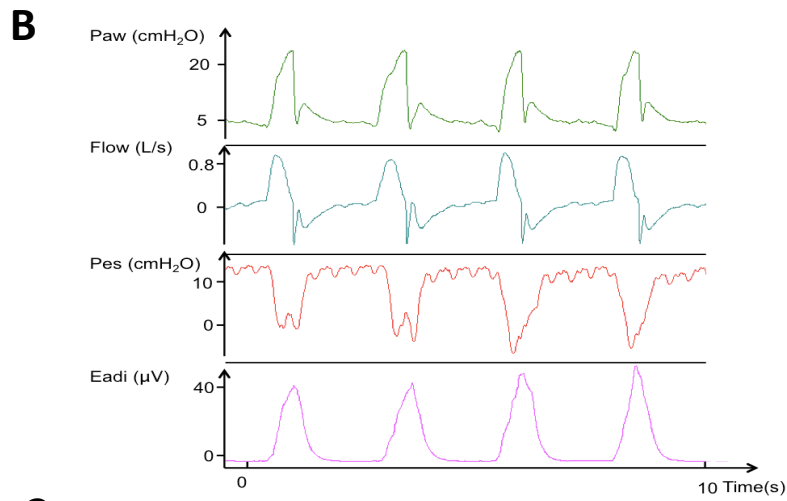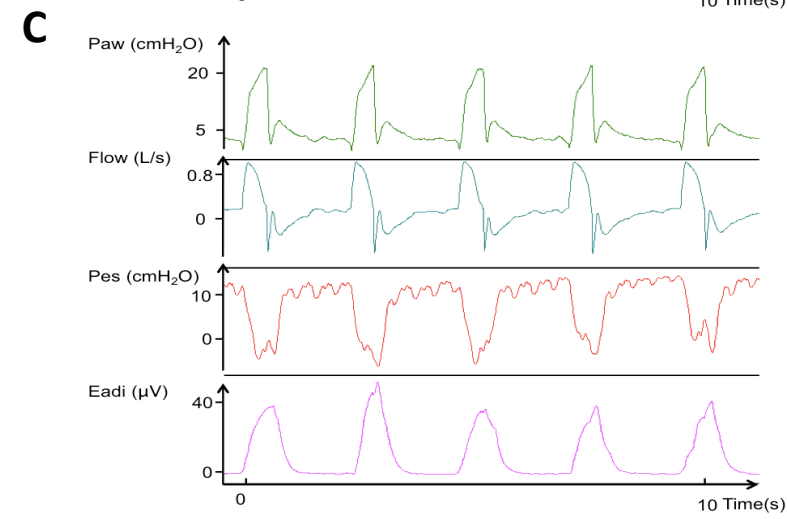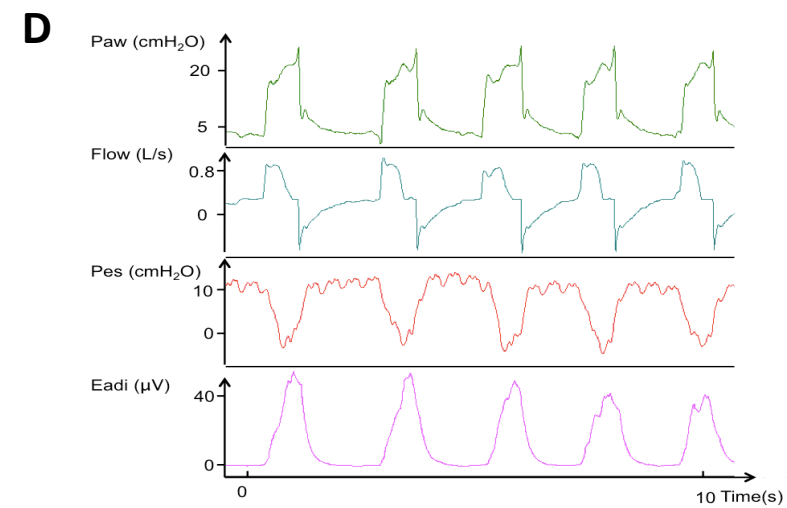

Supplement: Additional file 4: — Airway pressure (Paw), flow, esophageal pressure (Pes) and electrical activity of the diaphragm (Eadi) tracings during the four steps in a restrictive patient. A, step 1; B, step 2; C, step 3 and D, step 4. (PDF 261 kb) [file 13054_2017_1599_MOESM4_ESM.pdf]

**A**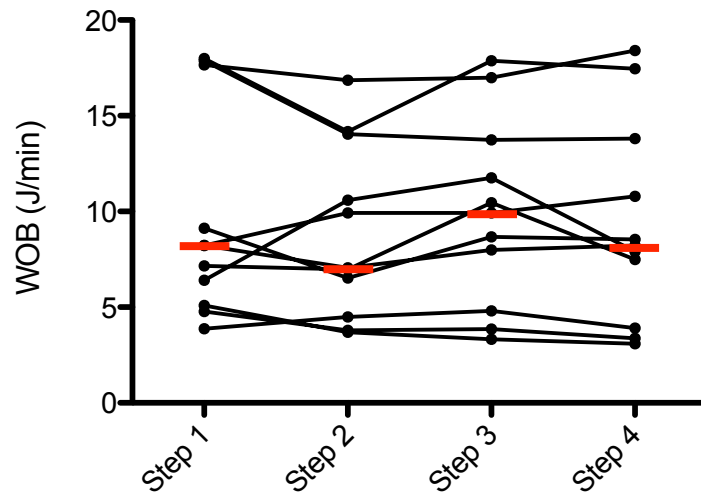**B**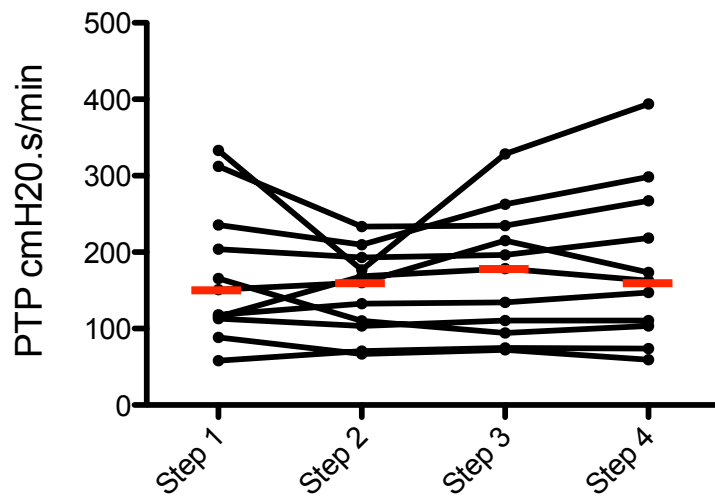

Supplement: Additional file 5: — Work of breathing (WOB) (A) and pressure time product (PTP) (B) during the four steps. Individual data. Horizontal red lines represent the median values. (PDF 45 kb) [file 13054_2017_1599_MOESM5_ESM.pdf]
